# Comparison of Affymetrix data normalization methods using 6,926 experiments across five array generations

**DOI:** 10.1186/1471-2105-10-S1-S24

**Published:** 2009-01-30

**Authors:** Reija Autio, Sami Kilpinen, Matti Saarela, Olli Kallioniemi, Sampsa Hautaniemi, Jaakko Astola

**Affiliations:** 1 Department of Signal Processing, Tampere University of Technology, Tampere, Finland; 2 Medical Biotechnology, VTT Technical Research Centre and University of Turku, Turku, Finland; 3 Institute for Molecular Medicine Finland (FIMM), University of Helsinki, Helsinki, Finland; 4 Computational Systems Biology Laboratory, Institute of Biomedicine and Genome-Scale Biology Research Program, University of Helsinki, Helsinki, Finland

## Abstract

**Background:**

Gene expression microarray technologies are widely used across most areas of biological and medical research. Comparing and integrating microarray data from different experiments would be very useful, but is currently very challenging due to the experimental and hybridization conditions, as well as data preprocessing and normalization methods. Furthermore, even in the case of the widely-used, industry-standard Affymetrix oligonucleotide microarrays, the various array generations have different probe sets representing different genes, hindering the data integration.

**Results:**

In this study our objective is to find systematic approaches to normalize the data emerging from different Affymetrix array generations and from different laboratories. We compare and assess the accuracy of five normalization methods for Affymetrix gene expression data using 6,926 Affymetrix experiments from five array generations. The methods that we compare include 1) standardization, 2) housekeeping gene based normalization, 3) equalized quantile normalization, 4) Weibull distribution based normalization and 5) array generation based gene centering. Our results indicate that the best results are achieved when the data is normalized first within a sample and then between-samples with Array Generation based gene Centering (AGC) normalization.

**Conclusion:**

We conclude that with the AGC method integrating different Affymetrix datasets results in values that are significantly more comparable across the array generations than in the cases where no array generation based normalization is used. The AGC method was found to be the best method for normalizing the data from several different array generations, and achieve comparable gene values across thousands of samples.

## Background

Microarray experiments have become an indispensable part of modern biological and biomedical research. As the number of studies using microarrays is growing all the time, it becomes increasingly important to compare and integrate data from multiple experiments and thereby improve the ability to make meaningful biological conclusions. Collections of microarray data from thousands of samples are emerging, but proper normalization methods are to a large extent lacking. To make optimal use of these datasets, improved methods for normalizing data from different studies in different laboratories are urgently required.

There are studies where gene expression data from different studies are systematically combined together. For example, computational models for defining modules in the transcriptional data [1-3] have been suggested. In addition, Oncomine, a database for gene expression data in cancer tissues including over 25,000 samples have been introduced [4,5]. Furthermore, a Celsius data warehousing system aggregates Affymetrix CEL-files and associated metadata [6]. These studies have included several thousands of samples from separate studies. Since different array types and normalization methods have typically been carried out for each study, the integration and direct comparison between the samples is difficult. Most of these meta-analyses are performed one-study-at-a-time, summing up the results together. There are also some publications describing the integration of data between different Affymetrix array generations. These methods are often based on the normalization of oligonucleotide microarray data using sequence overlaps between the individual oligos on the same slide [7-9]. However, the drawback of these approaches is that the non-overlapping probes need to be discarded. Therefore, particularly in the comparisons across multiple platforms, the number of informative genes is significantly reduced.

Here, our main objective was to test several known normalization methods for integrating gene expression values across thousands of experiments to be able to select a suitable method when combining datasets across Affymetrix array generations and experiment series. Even though the methods presented in this study are shown to work with the Affymetrix gene expression microarrays, they should be applicable also for integration experiments of other microarray platforms.

## Results

We compared and assessed the accuracy of five normalization methods for Affymetrix gene expression data using 6,926 Affymetrix experiments from five array generations. The methods that we compared include 1) standardization (Z), 2) housekeeping gene based normalization (HK), 3) equalized quantile normalization (Q), 4) Weibull distribution based normalization (WBL) and 5) array generation based gene centering (AGC). These were tested in the following ten combinations: Pure preprocessed data (MAS) without any further normalization, Z-, HK-, Q-, WBL-normalizations, and all of these normalization methods combined with the AGC method: MASAGC, ZAGC, HKAGC, QAGC and WBLAGC. The MAS, Z, HK, Q and WBL methods normalize the data within the samples, while the AGC method normalizes the data gene-wise between the samples.

Goodness of normalization can be measured in many ways. Here, we applied five different ways to estimate the degree of comparability between data from different array generations, including: 1) correlation between technical replicates, 2) correlation between randomly selected genes, 3) classification of the samples based on the anatomical classes, 4) comparison of correlations between the samples computed based on the anatomical classes and array generations, 5) stability of the house-keeping genes.

The data collection used in this study contained samples from Affymetrix array generations Hu6800 (HuGeneFL), HG-U95A, HG-U95Av2, HG-U133A, HG-U133 Plus 2. These array generations were selected as there were more than 500 samples hybridized on each of them in the database by the time of the comparison. At least half of the genes were in common between the array generations.

### Correlation between technical replicates

**Figure 1:**
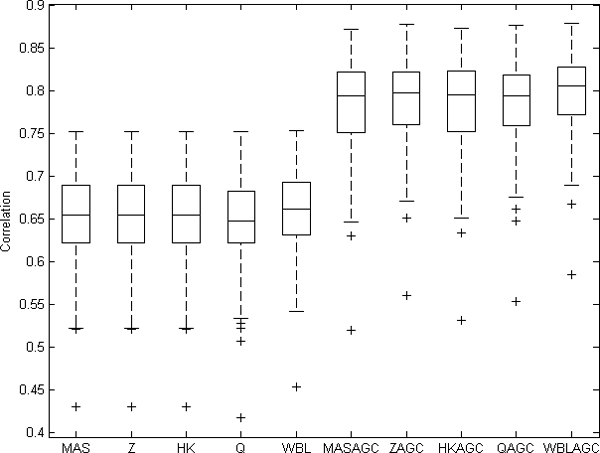
**Correlations between technical replicates**. Values from 132 technically replicated samples are normalized with different normalization methods and correlations are computed based on logarithmic values. Overall, the AGC improved the correlations.

The first metric for comparing the goodness of the normalization methods is to study the correlation between technical replicates. We have utilized an experiment series from St. Jude University [10,11] with 132 replicated RNA samples, each analyzed with both HG-U95Av2 and HG-U1331A. We calculated the correlations between these samples with each normalization method. This comparison method has been used in several studies in which data from different generations of Affymetrix arrays are combined and compared [7,8,12]. Here, the results are identical for MAS, Z and HK methods, since the correlation is linearly invariant. When comparing the methods without the AGC correction, the WBL gave the best results. We calculated the significance of the results using one-way ANOVA and performed the multiple comparison with Tukey's HSD. When AGC was merged with any of the normalization methods, the correlations increased significantly as compared with the first level normalization alone, with a significance level of *α *= 0.01. The WBLAGC gave the best results from the AGC methods, but the difference with the other AGC normalization methods was not significant (Figure 1).

### Correlation of randomly selected gene pair

Another method for comparing the goodness of the normalization methods is the comparison between the correlations of randomly selected genes [13]. Since it is unlikely that two randomly chosen genes are correlated with each other, the expected value for their correlation is zero. Now, the hypothesis is that *E(Corr*(*k*_1_, *k*_2_)) = 0 for genes *k*_1_, *k*_2 _where *k*_1 _≠ *k*_2_. The different array generations are known to induce some biases to the gene values that may further cause systematic errors in the data. These kinds of systematic array-wide variations may increase the correlation between randomly selected genes.

**Figure 2:**
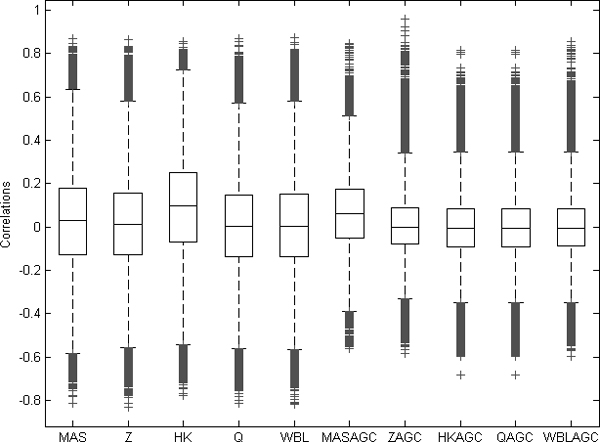
**Correlation values between randomly selected gene pairs**. The correlations were calculated between 500 randomly selected genes through 6,926 samples.  The four AGC-normalized datasets ZAGC, HKAGC, QAGC, and WBLAGC have mean values closer to zero than the others (ANOVA with multiple testing procedure, α < 0.01).

We selected randomly 500 genes that had values in each array generation and computed the correlations between each gene pair in the data normalized with the different methods. Further, we tested the mean values of the distributions of randomly selected correlations with one-way ANOVA and a utilized multiple testing procedure Tukey's HSD. The results showed that with significance level *α *= 0.01 the ZAGC, HKAGC, QAGC and WBLAGC had smaller mean values than the other methods. AGC correction was again found more robust than the other normalization methods, as these AGC-correction methods did not significantly differ from one another and were closer to zero (Figure 2).

### Samples to profiles classification

Third way to estimate goodness of normalization was the use of anatomical classes with the eVOC Anatomical System ontology [14]. An anatomical profile is the mean value of the logarithmic values of healthy samples of each tissue type. The profiles were calculated independently between the array generations. To obtain the profiles we used 1,464 samples from healthy tissues and cells including 15,931 genes from 35 anatomical classes. All the samples were annotated based on the eVOC Anatomical System ontology and the profile for the anatomical classes was created only if there were more than ten samples from that tissue. The 1,464 healthy samples were classified to the anatomical profiles with the nearest neighbour algorithm.

We used Pearson correlation as the metric in the classification. We computed the distance *d *= 1-*r*_*i*, *j*_, where *r*_*i*, *j *_is the correlation between the logarithmic values of the sample *i *and the profile *j*. Each sample was classified to the profile with the smallest distance. With the AGC normalized data the number of correctly classified samples increased substantially (Table 1). Obviously, there will always be some biological variability within a tissue, as well as sampling errors, methodological variability, and lab-to-lab variability that will render 100% classification accuracy unattainable. Nevertheless, the significant improvement of classification accuracy again testifies for the value of the AGC-based normalization methods.

**Table 1:** Results of the samples to profiles classification. 1,464 samples from healthy tissues and cells including 15,931 genes from 35 anatomical classes were classified. Anatomical classes, number of samples and number of different array generations within each of the classes are listed in the table. The percentages of correctly classified samples are calculated with each normalization method.

	**# samples**	**# array gens**	**MAS**	**Z**	**HK**	**Q**	**WBL**	**MASAGC**	**ZAGC**	**HKAGC**	**QAGC**	**WBLAGC**
**TOTAL**	**1464**	**5**	**74.2%**	**77.9%**	**75.5%**	**77.6%**	**76.1%**	**86.7%**	**89.6%**	**89.7%**	**89.8%**	**89.3%**

Aorta	24	1	100%	100%	100%	100%	100%	100%	100%	100%	100%	100%

Bronchus	94	2	81.9%	81.9%	81.9%	81.9%	81.9%	86.2%	86.2%	86.2%	86.2%	86.2%

Lung	117	4	47.9%	46.2%	46.2%	46.2%	46.2%	91.5%	88.0%	89.7%	86.3%	83.8%

Alveolus	34	1	100%	100%	100%	100%	100%	100%	100%	100%	100%	100%

Bone marrow	104	3	53.8%	53.8%	53.8%	52.9%	52.9%	86.5%	84.6%	86.5%	85.6%	86.5%

Peripheral blood	260	3	53.1%	70.8%	61.2%	70.8%	61.2%	62.7%	82.3%	82.3%	83.5%	82.3%

Tonsil	16	2	100%	100%	100%	100%	100%	100%	100%	100%	100%	100%

Colon	18	3	88.9%	88.9%	88.9%	88.9%	88.9%	88.9%	94.4%	88.9%	94.4%	88.9%

Liver	12	3	100%	100%	100%	100%	100%	100%	100%	100%	100%	100%

Pancreas	21	4	76.2%	81.0%	81.0%	76.2%	81.0%	95.2%	95.2%	95.2%	95.2%	95.2%

Kidney	49	3	73.5%	75.5%	73.5%	73.5%	75.5%	89.8%	87.8%	89.8%	87.8%	87.8%

Testis	13	3	92.3%	92.3%	92.3%	92.3%	92.3%	92.3%	92.3%	92.3%	92.3%	92.3%

Prostate	70	4	80.0%	75.7%	80.0%	75.7%	75.7%	98.6%	95.7%	98.6%	95.7%	95.7%

Foreskin	30	2	100%	100%	100%	100%	100%	100%	100%	100%	100%	100%

Ovary	14	2	57.1%	64.3%	57.1%	64.3%	57.1%	78.6%	78.6%	78.6%	78.6%	78.6%

Uterus	12	3	83.3%	75.0%	83.3%	75.0%	83.3%	100%	91.7%	100%	91.7%	91.7%

Endometrium	12	1	50.0%	83.3%	50.0%	66.7%	83.3%	100%	100%	100%	100%	100%

Placenta	28	3	67.9%	67.9%	64.3%	67.9%	67.9%	82.1%	75.0%	75.0%	75.0%	75.0%

Breast	25	4	64.0%	64.0%	64.0%	64.0%	64.0%	84.0%	96.0%	84.0%	96.0%	100%

Thyroid	16	2	87.5%	100%	88%	100%	100%	100%	93.8%	93.8%	93.8%	87.5%

Thymus	12	2	66.7%	75.0%	75.0%	75.0%	75.0%	83.3%	75.0%	83.3%	83.3%	83.3%

Muscle	11	2	90.9%	100%	90.9%	100%	100%	54.5%	54.5%	54.5%	54.5%	54.5%

Skin cuticle	85	3	91.8%	91.8%	91.8%	91.8%	91.8%	96.5%	97.6%	97.6%	97.6%	97.6%

Brain	35	3	54.3%	60.0%	54.3%	60.0%	60.0%	88.6%	88.6%	85.7%	85.7%	85.7%

Cerebral cortex	34	1	55.9%	61.8%	55.9%	61.8%	58.8%	58.8%	61.8%	52.9%	61.8%	58.8%

Frontal lobe	33	1	93.9%	93.9%	93.9%	93.9%	93.9%	93.9%	93.9%	93.9%	93.9%	93.9%

Hypothalamus	25	1	100%	100%	100%	100%	100%	100%	96.0%	96.0%	96.0%	96.0%

Cerebellum	33	2	84.8%	84.8%	84.8%	84.8%	84.8%	97.0%	97.0%	97.0%	97.0%	97.0%

Lens	11	1	100%	100%	100%	100%	100%	100%	100%	90.9%	100%	100%

Optic nerve	44	1	93.2%	93.2%	93.2%	93.2%	93.2%	93.2%	93.2%	93.2%	93.2%	93.2%

Striated skeletal muscle	92	2	100%	100%	100%	100%	100%	100%	100%	100%	100%	100%

Umbilical vein	38	1	97.4%	97.4%	97.4%	97.4%	97.4%	97.4%	97.4%	97.4%	97.4%	97.4%

Intervertebral disc	11	1	100%	100%	100%	100%	100%	100%	100%	100%	100%	100%

Ventricle myocardium	18	2	61.1%	61.1%	61.1%	61.1%	61.1%	100%	100%	100%	100%	100%

Atrium myocardium	13	1	100%	100%	100%	100%	100%	76.9%	76.9%	84.6%	76.9%	76.9%

### Correlations between samples from the same anatomical class

The correlation between samples from same anatomical class should be high indicating the similarity of samples in question. However, often the experimental conditions, preprocessing and array generation may cause high correlation, even if samples have very little in common based on the anatomy. This causes problems if the technical details of the experiments have more effect on the final data than the biological properties of the samples.

We assume that the expected value of correlation between the samples from same anatomical class is higher than the expected value of correlation of samples from different anatomical class, even if the samples were from the same experiment series or from same array generation. We calculated the correlations of gene expression levels between all the 1,464 healthy samples in the dataset and analyzed the values of them. Based on the array generation and the anatomy of samples, we divided these correlations into two groups: 1) Correlations from healthy samples from the same array generation but from different anatomical class, and 2) Correlations of healthy samples from the same anatomical class done with different array generations.

**Figure 3:**
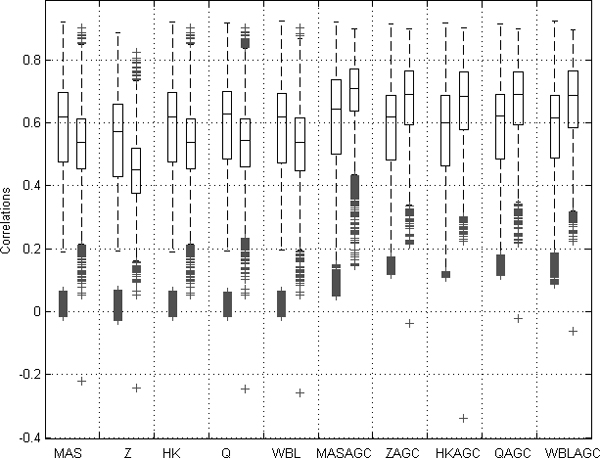
**Boxplot of distributions for two sets of correlations from data normalized with each normalization method**. The left values are correlations between samples from same array generation within different anatomical class. The right values are correlations between samples within the same anatomical class but with different array generation. All the 1,464 healthy samples were used in this comparison. When the AGC method was used, the mean value of the correlations between samples from the same anatomical class but different array generations were significantly higher than the mean of the correlations between the samples from same array generation but different anatomical class.

When AGC was not used, the array generation was superior to biological origin in defining the identity of the sample. In such cases the correlation between samples from the same array generations was significantly higher than the correlations between samples from the same anatomical class. When the data were AGC normalized, the correlations from the same anatomical origin were significantly higher than the correlations from different anatomical classes within the same array generation. The significance was tested with one-way ANOVA and multiple comparisons performed with Tukey's HSD with α < 0.01. As evidenced by the significance analysis, the AGC-normalization method reduced noise due to different array generations (Figure 3).

### Stability of housekeeping genes

If the data are normalized properly, the housekeeping gene values should be stable between experiments. This is based on the assumption that the housekeeping genes are expressed similarly in all samples across the array generations and tissues. However, it is known that the array generation can impact also on values for housekeeping genes, and the expression values of housekeeping genes in our material also seemed to differ based on the array generation.

We investigated the effects of different normalization methods on similarity of distributions of housekeeping genes from different array generations. The housekeeping genes under consideration were the same ones than used in the HK-normalization. The similarities were quantified with the Kullback-Leibler measure. We assumed that the values for each housekeeping gene from one array generation should be distributed similarly with the distribution of the gene across all the array generations. We divided the range of the gene value into 50 bins so that within each bin there are 2% of the gene values of the gene:

*a*_*j *_= *f*([0, 0.02, 0.04,..., 0.98, 1]),

where *f *is the empirical cumulative density function of the gene. We define *D *to be a set of all expression values measuring the expression of gene *k *and array generations to *A*_1_,..., *A*_*p*_. Now, for every array generation *i*, *D*_*i *_⊆ *D *is a set of all expression values of the housekeeping gene that are measured with the array generation *A*_*i*_. The probability density function of distribution *Q *for each array generation *i *is constant:

$$Q_{i}=\frac{\#\left(D_{i}\right)}{\#\left(D\right)},$$

where #(*D*) is the number of values in the data set *D*. We assume that the data values within each percentile group are distributed along this constant distribution. We compute the discrete distribution of the gene values from all array generations within each of these percentiles of the data:

$$P_{i,j}=\frac{\#\left(\left\{a_{j}\leq x<a_{j+1}|x\in D_{i}\right\}\right)}{\#\left(\left\{a_{j}\leq y<a_{j+1}|y\in D\right\}\right)},$$

where *i *is the array generation and *j *the percentile group. Thus, it is assumed that the distribution *P *is similar with the distribution *Q*. The distance between these distributions for each array generation *i *is calculated with the Kullback-Leibler distance:

$$d_{i}=\sum_{j}P_{i,j}\log\left(\frac{P_{i,j}}{Q_{i}}\right),$$

where *j *goes through the percentiles. The smaller the distance is between the distributions, the closer the distributions are to each other.

**Figure 4:**
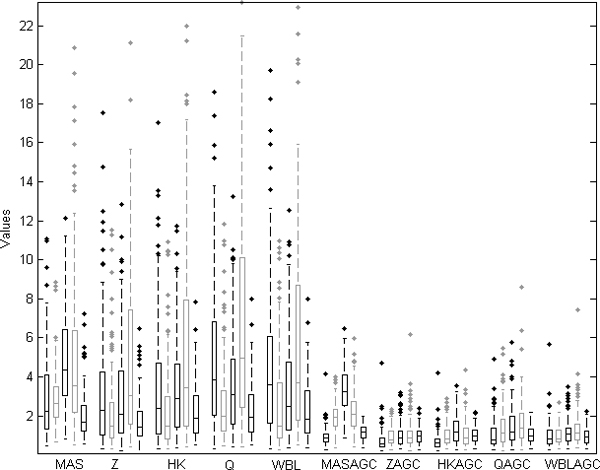
**The boxplot of the Kullback-Leibler distances with each array generation and normalization**. The KL-distances of 126 housekeeping genes from each array generation from all 6,926 samples were computed. Each boxplot includes the KL-distances of the 126 genes. The array generations are ordered within each of the normalization methods in chronological order of array generations; Hu6800, HG-U95A, HG-U95Av2, HG-U133A and HG-U133 Plus 2.0.

We calculated these distances for each of the 126 housekeeping genes [15] from each array generation. The AGC method greatly reduced the distance between gene values from one array generation and gene values from all array generations (Figure 4).

## Discussion

An important step to integrate Affymetrix data is to develop methods that result in comparable values for a wide spectrum of array generations. Further, it is crucial to use different measures for goodness of the normalization as the objectives for the normalization may vary between studies. The methods we have developed will significantly facilitate data comparisons across thousands of samples, with minimal loss of informative genes, which was a serious limitation in earlier studies.

We have applied five different normalization methods for Affymetrix gene expression data. The array-generation based gene centering method (AGC) [16] can be merged together with any within-slide normalization method. Here, we tested the values normalized with the AGC method combined with five different normalization methods and observed significantly improved results. All the normalization methods compared here are based on different assumptions and therefore also the effect on normalization strategies may vary. The traits of the normalization methods are collected into Table 2.

**Table 2:** The properties of the normalization methods. MAS, Z, HK, Q, and WBL are sample-wise normalization and the gene-wise method AGC can be merged to each of them. This table lists the properties of each method.

	**MAS**	**Z**	**HK**	**Q**	**WBL**	**AGC**
**Sample-wise normalization**	Yes	Yes	Yes	Yes	Yes	No

**Gene-wise normalization**	No	No	No	No	No	Yes

**Considers the array generation**	No	No	No	No	Yes	Yes

**Includes scaling**	Yes	Yes	Yes	No	Yes	Yes

**Based on distribution**	No	No	No	Yes	Yes	No

**May change the order of the values within a sample**	No	No	No	No	No	Yes

We have employed five different criteria to measure goodness of the normalizations. The results showed that the AGC method improved the results systematically and that the AGC normalized data became comparable across the array generations, as suggested by the classification accuracy of different anatomical samples, and the improved correlation of the data from the same samples analyzed on two different array generations. The AGC method combined with the Q normalization is used in for almost 10,000 samples in GeneSapiens database [16,17].

## Conclusion

The gene expression data from 6,926 samples were analyzed together in order to find computationally effective and well-performing method to normalize a large number of the data samples to be directly comparable with each other. All the samples were measured with Affymetrix microarrays, but the various array generations hinder the comparability. Ten different combinations of five normalization method were utilized. The array generation based centering of gene values was found to perform the best, especially if utilized together with the equalized quantile normalization or WBL-normalization.

## Methods

### Data preprocessing

The data set of 6,926 Affymetrix arrays includes several different array generations with different probe sets. Further, the probe set values need to be converted to gene values. We took median of the normalized values from different probe sets that linked to the same ENSEMBL gene identifier [18] in order to have only one expression value for each gene.

As different preprocessing methods often complicate the data integration, we used data from which the raw data (CEL-files) were available. For all these experiments we used MAS5 preprocessing method with default parameters [19].

The selection of preprocessing method for Affymetrix gene expression data is a controversial topic, and although different opinions exist for optimal preprocessing method [20] in recent comparison studies MAS5 provided the most faithful cellular network construction [21] and optimal identification of differentially expressed genes [22]. In addition, in several studies [13,21,23] it has been stated that other preprocessing methods may also create false correlation between the samples.

### Expression value standardization (Z)

Gene value standardization is widely used method for normalizing the gene expression values. In standardization the logarithmic signal values of genes are normalized to have zero as mean or median and one as standard deviation:

$$\underline{z}=\frac{\underline{x}-\mu }{\sigma }$$

where the vector *x* consists of the logarithmic values of a sample, *μ *is the mean or median and *σ *the standard deviation of the sample. The standardized values are often called as Z-scores or median Z-scores. Here, Z refers to median Z-scores.

### Housekeeping gene centering (HK)

The housekeeping gene centering (HK-centering) scales the data using a scaling factor that is defined based on the housekeeping genes common with most popular array generations. The assumption behind the HK-centering is that the set of housekeeping genes is expressed identically across the samples. This assumption is found to be unrealistic in several settings [24]. However, even though the assumption that a gene set is constantly expressed across a wide spectrum of tissues may be unrealistic, there are genes that are relatively constant in one tissue type. Consequently, we have included the HK-centering in this study. The scaling factor is defined based on a limited set of housekeeping genes that are found from the most common array generations. First, a suitable set of HK-genes needs to be selected. We selected a set of 126 genes that were found from most of the Affymetrix array generations [15]. Next, the target intensity (TI) value for the gene set is selected. Here, the target intensity value is computed as

$$TI=\frac{1}{n}\sum_{j=1}^{n}\left(avg({\underline{x}}_{j,HK})\right),$$

where x_*j*, *HK *_are the values of housekeeping genes in sample *j*. The gene values of each sample can be calculated with

$$\underline{y}=\frac{TI}{avg({\underline{x}}_{HK})}\underline{x},$$

where *x* are the expression values of the sample.

### Equalized quantile normalization (Q)

In several cases it is desirable to scale the samples so that the minimum and the maximum values are the same order of magnitude. Further, often down-stream analysis methods assume that at least standard deviations or means of the values are equal. Therefore, we have utilized equalized quantile normalization (Q) algorithm to normalize the data [25].

In the basic quantile normalization all samples are normalized to have the same distribution [26-28]. This distribution is the mean distribution of all samples in analysis. Therefore, the quantile normalization requires the same number of values in each sample and hence, the quantile normalization is not directly usable for normalizing expression data from different array generations.

We utilized the equalized quantile normalization (Q) that constructs a data set having the desired distribution that has been determined prior to transformation. In the Q-normalization the assumptions are the same as in quantile normalization; the sorted order of the data values should not be changed by the normalization method and the logarithmic signal values can be presented with a predefined distribution. This distribution is the same for every sample through all array generations.

**Figure 5:**
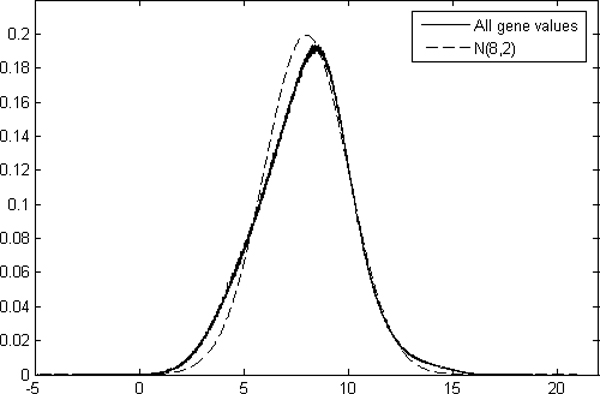
**Distribution of the gene values and the normal distribution**. The normal distribution with mean 8 and standard variation 2 is near to the distribution of logarithmic values of all 6,926 samples from five different array generations.

In this study, we evaluated the distribution of the logarithmic MAS5 probe set values for every Affymetrix array generation and found that we can approximate the signals by equalizing the signal-log-values to normal distribution. The distribution of logarithmic values from all samples (N = 6,926) was very near to the normal distribution with mean value 8 and standard deviation 2 (Figure 5). Accordingly, we use N(8,2) normal distribution as the target distribution for the probe set values of the samples.

### Weibull distribution based normalization (WBL)

We have also used the Weibull distribution based normalization (WBL), a way to normalize and correct Affymetrix microarray probe set data [29]. In the Weibull distribution based normalization method it is assumed that the logarithmic probe set values can be adjusted based on the parameters of the Weibull distribution. In order to obtain comparable data, each sample is corrected to have the same shape and scale parameters as the corresponding array generation has. Depending on the array generation, the scale and shape parameters of the Weibull distribution may still differ a bit after normalization, but they are the same for every sample within the same array generation.

For each array generation we collected the array-generation data that are a group of samples analyzed using the MAS5 with default parameters [19,29]. These samples were selected since they represent the distribution of all the samples done with the same array generation. These were used as comparison material and the parameters of these data were set to be the default parameters for each array generation. Based on these data we computed the Maximum Likelihood (ML) estimates [30] for the shape parameter *β*_*i *_and the scale parameter *η*_*i *_for each array generation *i*. The scale parameter varied from 8.19 to 8.57, while the shape parameter varied from 3.11 to 3.85. The ML-estimates were calculated for each sample and probe set values were adjusted in order to have same parameter estimates for each of the samples within an array generation. For the sample *j *this normalization can be done with the formula:

$${\underline{y}}_{j}={\hat{\eta }}_{i}{\hat{\eta }}_{j}^{-\left({\hat{\beta }}_{j}/{\hat{\beta }}_{i}\right)}{\underline{x}}_{j}^{\left({\hat{\beta }}_{j}/{\hat{\beta }}_{i}\right)}$$

where *η*_*i *_and *β*_*i *_are the ML-estimates for scale and shape parameters in the array generation *i*, and *η*_*j *_and *β*_*j *_are the ML-estimates for the scale and shape parameters in the sample *j*. Finally, the WBL-normalized gene values were set to be the median values of the probe sets linked to each gene.

### Array generation based gene centering (AGC)

In the AGC method we assume that the mean of expression values of one gene in each array generation should be the same. If the mean value of some of the array generations differs substantially from the others, the shift is assumed to be caused by the array generation based variation. The AGC method aims to correct this variation.

The AGC method requires the collection of samples to be relatively large so that one can assume the distribution of logarithmic values of each gene *k *to represent the total distribution of all potential expression values across all tissues in that array generation *i*. Therefore, the AGC normalization method normalizes the data to have the mean values *μ*_*i*, *k *_= *μ*_*k *_for all array generations *i*, where *μ*_*k *_is the mean of all logarithmic values of the gene *k*. We assume that the minimum and the maximum estimates for the gene value are reached and the range of the gene *k *should approximately be [*a*_*k*_, *b*_*k*_], where *a*_*k *_is the lowest 2% value and *b*_*k *_is the largest 2% value of gene *k*. After array-generation based centering none of the values should go over this range. However, if the new centered value exceeds the range, the difference is diminished towards the range limits with coefficient *c*, 0 ≤ *c *≤ 1. Here, the coefficient is set to *c *= 1/5 in order to diminish the greatest and smallest values. The centered values can now be obtained with

$${\hat{x}}_{k,j}=\log_{2}(x_{k,j})-(\mu_{k,i}-\mu_{k})$$

where *x*_*k*, *j *_is the value of gene *k *in sample *j *measured with the array generation *i*, *μ*_*k*, *i*_is the mean of the logarithmic values of gene *k *across array generation *i *and *μ*_*k *_is the mean of the logarithmic values of gene *k *across all array generations. Further, the resulted AGC values are adjusted based on the equation

$$AGC\_Value_{k,j}=\left\{ \begin{array}{l} b_{k}+c({\hat{x}}_{k,j}-b_{k}),\text{ for }{\hat{x}}_{k,j}>b_{k},\\ a_{k}-c(a_{k}-{\hat{x}}_{k,j}),\text{ for }{\hat{x}}_{k,j}<a_{k}\\ {\hat{x}}_{k,j},\text{otherwise}\text{. } \end{array}\right.,$$

Finally, the values are converted back to the original scale by

$$y_{k,j}=2^{AGC\_Value_{k,j}}.$$

**Figure 6:**
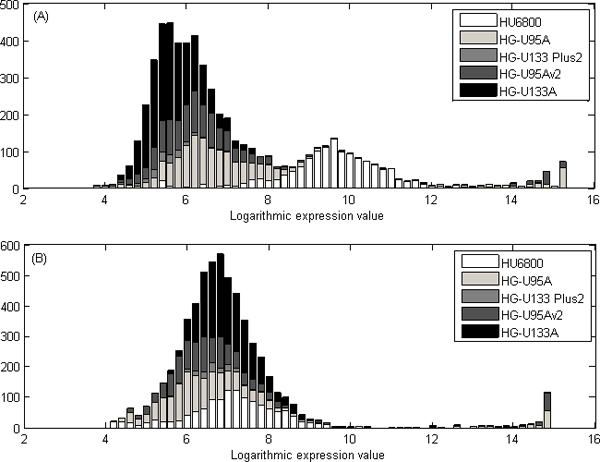
**Illustration of the effect of AGC normalization for a prostate specific gene *KLK3***. (A) The logarithmic expression values of the *KLK3 *gene before the AGC normalization are distributed based on the array generation. (B) The values are normalized with the AGC method, and there is no significant difference between the values from different array generations. The figure illustrates prostate specific gene, *KLK3*, and gene values in prostate samples have great values both before and after normalization. After AGC-normalization, the large values not caused by anatomy but array generation HU6800 are diminished.

The AGC method can be used together with any of the within slide methods presented above. After the AGC normalization the mean values of distributions of array generations are centered to have the same mean (Figure 6).

We have utilized equalized quantile normalization Q combined with the AGC method in the GeneSapiens database [16] with almost 10,000 samples in [17]. There are also few other methods [31-33] used to combine different datasets. However, these are computationally demanding and therefore impractical to use for a dataset including thousands of samples.

## Competing interests

A patent application of the AGC normalization protocol has been filed.

## Authors' contributions

RA, SK and MS contributed to the development and testing of the normalization. SH contributed to the development of normalization and supervised the comparison and validation of the normalization methods. JA and OK supervised the project. RA wrote the manuscript. All authors read and approved the final manuscript.
